# Exploratory Study on the Methodology of Fast Imaging of Unilateral Stroke Lesions by Electrical Impedance Asymmetry in Human Heads

**DOI:** 10.1155/2014/534012

**Published:** 2014-05-29

**Authors:** Jieshi Ma, Canhua Xu, Meng Dai, Fusheng You, Xuetao Shi, Xiuzhen Dong, Feng Fu

**Affiliations:** Department of Biomedical Engineering, Fourth Military Medical University, Xi'an 710032, China

## Abstract

Stroke has a high mortality and disability rate and should be rapidly diagnosed to improve prognosis. Diagnosing stroke is not a problem for hospitals with CT, MRI, and other imaging devices but is difficult for community hospitals without these devices. Based on the mechanism that the electrical impedance of the two hemispheres of a normal human head is basically symmetrical and a stroke can alter this symmetry, a fast electrical impedance imaging method called symmetrical electrical impedance tomography (SEIT) is proposed. In this technique, electrical impedance tomography (EIT) data measured from the undamaged craniocerebral hemisphere (CCH) is regarded as reference data for the remaining EIT data measured from the other CCH for difference imaging to identify the differences in resistivity distribution between the two CCHs. The results of SEIT imaging based on simulation data from the 2D human head finite element model and that from the physical phantom of human head verified this method in detection of unilateral stroke.

## 1. Introduction

Stroke refers to the rapid loss of brain function because of a disturbance in blood supply to the brain. Stroke can be classified into two major categories: ischemic and hemorrhagic. Related studies show that the disease is the second leading cause of death in the world [1]. The three-year cumulative incidence could reach 16.8 per 1000 [2]. Thirty-day mortality could reach 20% [3], and the disability rate is higher than 65% [4]. The timely detection and treatment of stroke reduces the risk of death of patients [5] and is important in improving the prognosis [6].

The devices utilized to diagnose stroke include CT and MRI, which are noninvasive means of detection involving images of high spatial resolution. However, these devices are bulky and expensive and are available only in well-equipped hospitals. Community hospitals generally do not have such devices. Hence, a portable technique for the detection of stroke is needed to preliminarily diagnose sudden stroke cases in community hospitals.

Biological tissues are characterized by electrical properties (conductivity and permittivity) that allow electrical current to flow in the presence of an electric field [7]. Electrical properties depend on the constituent elements and structure of tissue; therefore, each type of human tissue and body fluids is with specific conductivity and permittivity. Thus, measurements of electrical impedance can be used to differentiate between different types of tissues or to assess the state of tissue [8]. Changes in the composition and structure of a tissue modify its electrical properties and consequently change its electrical impedance. The occurrence of stroke can make such changes and modify the electrical impedance distribution in the human head.

Based on the fact that different biological tissues have different electrical properties, electrical impedance tomography (EIT), also known as bioimpedance tomography [9], applies a safe alternating current to the body surface, measures body surface potential, and reconstructs images of resistivity distribution or its change within the body through a certain image reconstruction algorithm. Considering its portability, noninvasiveness, and the absence of ionizing radiation, EIT has been studied as a monitoring and assessment tool for medical applications, such as detecting breast cancer [10], assessing abdominal hemorrhage [11], imaging lung ventilation [12], and assessing intracranial impedance variation [13, 14]. Thus, EIT has potential for detection of stroke [15].

EIT imaging approaches include time difference EIT (tdEIT) or dynamic EIT, static EIT, and frequency difference EIT (fdEIT) [13]. Difference imaging is conducted between two frames of EIT data measured at two time points in tdEIT. The distribution of the absolute resistivity value in the measured objects with one frame of EIT data is reconstructed in static EIT and difference imaging between two frames of EIT data measured at two frequencies is conducted in fdEIT. In theory, static EIT satisfies the requirements of reconstructing an image of a stroke lesion at a single time. However, the method has been limited by fundamental ill-posedness as well as technical difficulties due to a limited amount of measurements, unknown boundary geometry, and uncertainty in electrode positions, while systematic measurement artifacts and random noise are also the limitations of the method [16]. In principle, fdEIT also satisfies the requirements of reconstructing an image of a stroke lesion [17]. However, fdEIT requires high-performance hardware systems and imaging algorithms [16–19]. fdEIT is in the stage of system optimization and algorithm improvement and can only image objects, such as carrots and bananas, in physical phantoms without skull.

Through extensive research, our research group established a dynamic EIT platform for monitoring cerebral impedance and utilized it to dynamically monitor the conditionally controllable progress of stroke in animal models [20, 21]. Given that dynamic EIT requires time-referenced data, it is inapplicable to cases where a single image in time is required or when time-referenced data are unavailable. Hence, dynamic EIT cannot be utilized to conduct rapid single-time imaging of presented stroke lesions. A rapid electrical impedance imaging method with no high demands on imaging systems should be investigated to rapidly diagnose stroke patients. Accordingly, the objective of this study was to establish a new EIT method to determine whether there was a lesion in a subject's head and estimate approximately the location and the size of the lesion.

The median sagittal plane of the brain separates the brain into two basically symmetrical hemispheres. Published reports have demonstrated that a stroke lesion is usually located in one cerebral hemisphere [22]. No significant difference in impedance exists between the two cerebral hemispheres of normal humans, but stroke significantly increases the difference [23–25]. The bilateral impedance of normal human heads is almost symmetrical; however, a stroke can alter such symmetry. Thus, a stroke may be detectable by measuring the electrical impedance asymmetry in the head of a stroke patient.

Given that unilateral stroke lesions often generate significant symptoms on the other parts of the body [26], this can be utilized to determine which craniocerebral hemisphere (CCH) with the lesion. Effective measurements to determine which CCH was damaged by unilateral stroke lesion were also studied [27, 28]. Thus, the damaged and undamaged CCHs can be determined in practice.

An EIT method called symmetrical electrical impedance tomography (SEIT) was then proposed to image the asymmetry of the bilateral impedance of human heads to detect unilateral cerebral lesions. In this technique, electrical impedance tomography (EIT) data measured from the undamaged craniocerebral hemisphere (CCH) is regarded as reference data for the remaining EIT data measured from the other CCH for difference imaging to identify the differences in resistivity distribution between the two CCHs. The technical validation of this approach in the rapid detection of unilateral cerebral lesions was verified with finite element simulation and physical phantom experiments.

## 2. Materials and Methods

### 2.1. Evaluation of Electrical Impedance Asymmetry in the Human Head with EIT Data

#### 2.1.1. The Symmetrical Relationship of EIT Data Measured from the Two Hemispheres of the Human Head

Craniocerebral EIT data refer to the boundary voltages (BVs) obtained by injecting the driving current to the human head by EIT. Differences in EIT data from the two CCHs can be utilized to assess the impedance asymmetry of the two CCHs caused by a unilateral lesion.

**Figure 1:**
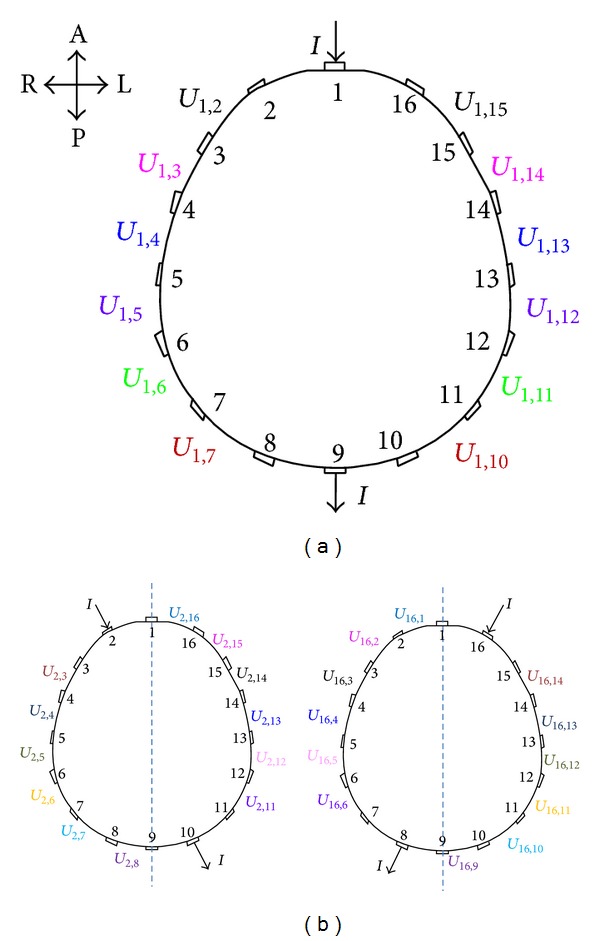
Illustrations of SBVP formation. (a) When the current was injected through electrode pair (1, 9), six groups of SBVP were formed by six BVs measured from the electrodes on the right hemisphere of the head and six other BVs from the left hemisphere. (b) The drives on electrode pair (2, 10) and (16, 8) constructed a pair of symmetrical drives. Six groups of SBVP were formed by six BVs measured from the right hemisphere during the drive on electrode pair (2, 10) and six BVs from the left hemisphere during the drive on electrode pair (16, 8). Similarly, another six groups of SBVP were formed by six BVs from the right hemisphere during the drive on electrode pair (16, 8) and six BVs from the left hemisphere during the drive on electrode pair (2, 10). This pair of symmetrical drives generated 12 groups of SBVP. Two BVs that formed one group of SBVP are marked with the same color (A: anterior; P: posterior; L: left; and R: right).

Considering that the EIT data-measuring pattern involves multiple measurements based on multiple polar drives [29], the BVs in one frame of EIT data acquired by a 16-electrode EIT system were marked as *U*_*i*,*j*_, where the subscript *i* is the drive number ranging from 1 to 16 and corresponding to the polar-drive electrode pairs (1, 9), (2, 10), (3, 11),…, (16, 8); the subscript *j* is the measurement number ranging from 1 to 16 and corresponding to the adjacent-measurement electrode pairs (1, 2), (2, 3), (3, 4),…, (16, 1). The positions and corresponding numbers of the 16 electrodes are shown in Figure 1. When the driving and data-measuring electrodes have a common electrode, *U*_*i*,*j*_ (e.g., *U*_1,1_) is considered as invalid data for EIT imaging. Sixty-four *U*_*i*,*j*_ were deemed invalid, and the remaining 192 *U*_*i*,*j*_ were considered valid EIT data to be used in subsequent EIT image reconstruction.

The EIT data from the two hemispheres utilized to evaluate craniocerebral impedance asymmetry were transformed into symmetrical boundary voltage pairs (SBVPs) as follows. For one frame of EIT data, two BVs measured on two pairs of data-measuring electrodes symmetrical to the median sagittal plane of the brain were defined as SBVP. SBVP was generated by anterior-posterior drive and symmetrical drives. An anterior-posterior drive refers to injecting the driving current through electrode pair (1, 9) or (9, 1). When electrode pair (1, 9) carried the current, six groups of SBVP were generated (Figure 1(a)); thus, the two anterior-posterior drives can generate 12 groups of SBVP. In addition, a pair of symmetrical drives refers to two drives, where the line joining one pair of driving electrodes and the line joining the other are symmetrical to the line joining electrodes 1 and 9. Examples include the drives on electrode pair (2, 10) and (16, 8), drives on electrode pair (3, 11) and (15, 7),…, drives on electrode pair (8, 16) and (10, 2). Seven pairs of symmetrical drives were obtained. In one pair of symmetrical drives, such as the drives on electrode pair (2, 10) and (16, 8), 12 groups of SBVP were generated (Figure 1(b)). Thus, seven pairs of symmetrical drives generated 84 groups of SBVP. Overall, valid data in one frame of EIT data can be divided into 96 SBVP groups, containing 192 BVs. Among them, 96 BVs *U*_*i*,*j*_ (*j* = 1,…, 8; *i* = 1,…, 16) measured from the right hemisphere of the head (Tables 2, 3, 4, 5, 6, 7, 8, and 9, first row) had a relationship with the remaining 96 BVs *U*_*i*′,*j*′_ (*j*′ = 16, 15, 14, 13, 12, 11, 10, 9; *i*′ = 1, 16, 15, 14, 13, 12, 11, 10, 9, 8, 7, 6, 5, 4, 3, 2) measured from the left hemisphere of the head (Tables 2–9, second row). The numbers of SBVPs are shown in the third row of Tables 2–9.

#### 2.1.2. Evaluation of the Electrical Impedance Asymmetry

As mentioned above, 192 BVs in one frame of EIT data were divided into 96 groups of SBVP. Each group contained two BVs measured from two symmetrical data-measuring electrode pairs. Their numerical difference reflected the impedance asymmetrical degree of symmetrical regions in the two CCHs. The difference was defined as the index of asymmetry (IA) and was calculated by

(1) $$\begin{array}{c} \text{IA}=100\times \frac{\left|L-R\right|}{\left(L+R\right)/2}\%. \end{array}$$

In (1), *L* and *R* are the two BVs in one group of SBVP and are measured from the left and right CCHs, respectively.

When a small difference exists in the two BVs in one group of SBVP, IA is close to 0 and the asymmetry level of these two impedance data is relatively low and vice versa. The maximum value of IA (IA_max⁡_) corresponds to the highest level of impedance asymmetry of the two hemispheres of the head and decides the upper limit of the range of IA. Therefore, IA_max⁡_ was utilized as the index to evaluate the asymmetry of EIT impedance data measured from the two hemispheres of the head.

### 2.2. Symmetrical EIT (SEIT)

SEIT is a difference EIT method, within which one frame of EIT reference data **V**_ref_ was first constructed based on one frame of EIT raw data **V**_cur_ and changes of **V**_cur_ with respect to **V**_ref_ were then identified. Therefore, SEIT mainly involves two main procedures: constructing SEIT reference data and reconstructing the SEIT image.

#### 2.2.1. Construction of SEIT Reference Data

A frame of EIT raw data can be divided into two parts measured from the left and right CCHs. Half of a frame of EIT raw data measured from the undamaged CCH can be utilized to construct SEIT reference data. The undamaged CCH is referred to as the “reference hemisphere.”

**Figure 2:**
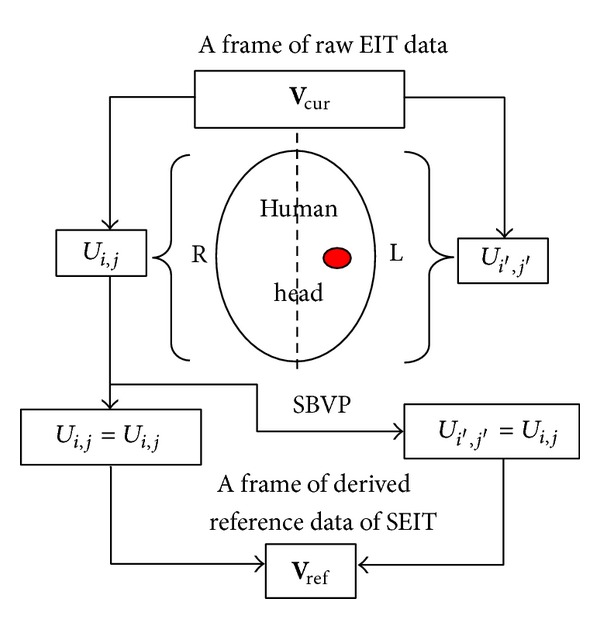
Construction of SEIT reference data. During construction, the raw EIT data measured from the undamaged CCH (e.g., the right CCH) was copied to the contralateral side according to the relationship of SBVP.

In this research, the CCH without anomaly was regarded as the reference hemisphere. SEIT reference data were constructed as follows. Firstly, 192 valid BVs of a frame of EIT raw data **V**_cur_ were divided into two parts: 96 BVs *U*_*i*,*j*_ (*j* = 1,…, 8; *i* = 1,…, 16) were measured from the right (undamaged) CCH and the other 96 BVs *U*_*i*′,*j*′_ (*j*′ = 16, 15, 14, 13, 12, 11, 10, 9; *i*′ = 1, 16, 15, 14, 13, 12, 11, 10, 9, 8, 7, 6, 5, 4, 3, 2) were measured from the left (damaged) CCH. This procedure revealed the relationship of SBVP between *U*_*i*′,*j*′_ and *U*_*i*,*j*_. Secondly, a frame of SEIT reference data **V**_ref_ with all zero values was constructed, and the 96 BVs *U*_*i*,*j*_ measured from the undamaged CCH were duplicated and stored into the same positions of **V**_ref_. These BVs were also stored into the positions of the 96 BVs *U*_*i*′,*j*′_ of **V**_ref_ according to the relationship of SBVP (Tables 2–9). A frame of SEIT reference data **V**_ref_ containing 192 valid BVs was then constructed. The construction of SEIT reference data is shown in Figure 2.

With the undamaged CCH as the reference hemisphere, the object of SEIT image reconstruction is to solve the resistivity distribution change in the damaged CCH with respect to that in the reference hemisphere. Thus, a stroke lesion was expected to be observed in the damaged side.

#### 2.2.2. SEIT Image Reconstruction

SEIT image reconstruction is similar to general difference EIT imaging. When a frame of SEIT reference data **V**_ref_ was constructed, the damped least squares image reconstruction algorithm [30] was utilized for difference EIT image reconstruction. According to the changes in EIT boundary voltages (Δ**V**), difference EIT image reconstruction solved the changes in the internal resistivity of the measured subject with

(2) $$\begin{array}{c} \Delta \rho =B\Delta V. \end{array}$$

**Figure 3:**
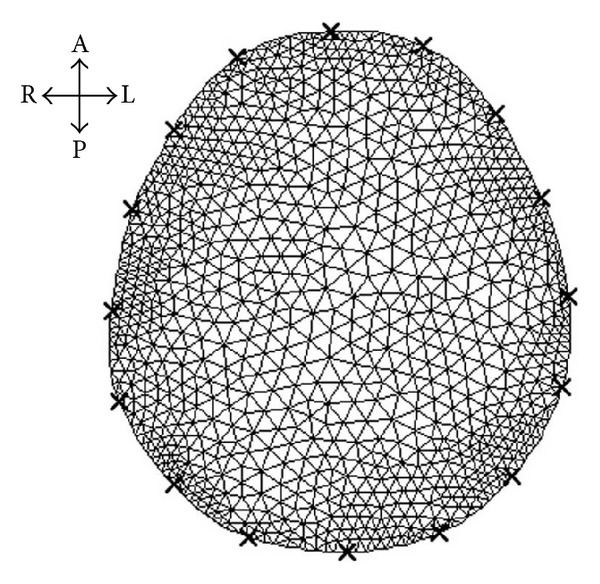
Image reconstruction model. The image reconstruction model consisted of 1804 triangular elements, 984 nodes, and 16 electrodes. The model was used for image reconstructions in all imaging experiments, including simulation and physical phantom experiments (A: anterior; P: posterior; L: left; and R: right).

In (2), matrix **B** is the image reconstruction matrix calculated from sensitivity coefficient matrix **S** [30]. Matrix **S** is the linearized sensitivity matrix, and its elements reflect the relationship between the resistivity changes in the finite elements of the imaging region and the changes in the EIT boundary voltages. Matrix **S** was acquired by solving EIT forward problems [30] with an image reconstruction model (Figure 3). The changes in EIT boundary voltages were calculated according to

(3) $$\begin{array}{c} \Delta V=\frac{V_{\text{cur}}-V_{\text{ref}}}{V_{\text{ref}}}. \end{array}$$

During image reconstruction, the image reconstruction software directly employed the reconstruction model and image reconstruction matrix **B** to calculate (2) and to visualize the reconstructed image.

Each pixel in the reconstructed image was with a single value of relative resistivity change (Δ*ρ*) in arbitrary units (AU), and the values were mapped to a colorbar showing the change of resistivity distribution. EIT image was displayed using a false color mapping, and the mapping index *g*(*x*, *y*) of the pixel (*x*, *y*) was calculated according to the following [31]:

(4) $$\begin{array}{c} g\left(x,y\right)=\frac{\Delta \rho \left(x,y\right)+{\Delta \rho }_{\max}}{2{\Delta \rho }_{\max}}, \end{array}$$

in (4) Δ*ρ*(*x*, *y*) is the resistivity change at the pixel coordinates (*x*, *y*) in the SEIT image. Δ*ρ*_max⁡_ is the maximum absolute value of Δ*ρ*(*x*, *y*). After the mapping index *g*(*x*, *y*) was calculated, the color of the pixel (*x*, *y*) was determined according to Table 1 [31].

In the color map, red indicated a decrease in resistivity distribution while blue indicated the opposite change.

#### 2.2.3. Analysis of Reconstructed SEIT Images

To quantitatively analyze the pixel intensities in the abnormal resistivity distribution region in the reconstructed image, the mean abnormal resistivity value (MARV), which is the absolute value of the average of abnormal resistivity for all of the pixels in the region of interest (ROI), is calculated as

(5) $$\begin{array}{c} \text{MARV}=\left|\frac{1}{N}\sum \Delta \rho \left(x,y\right)\right|\;xy\in \text{ROI}, \end{array}$$

where Δ*ρ*(*x*, *y*) is the resistivity change at the pixel coordinates (*x*, *y*) in the SEIT image and *N* is the total number of the pixels within ROI, which is determined by a preset threshold defined by

(6) $$\begin{array}{c} \frac{\Delta \rho_{\max}-\left|\Delta \rho \left(x,y\right)\right|}{\Delta \rho_{\max}}\leq t. \end{array}$$

In the above formula, Δ*ρ*_max⁡_ is the maximum absolute value of Δ*ρ*(*x*, *y*) and *t* is the threshold parameter. Previous studies have indicated that 0.2 is an appropriate value for *t* [20].

### 2.3. SEIT Imaging Based on Simulation Data

#### 2.3.1. Finite Element Modeling and Simulation of EIT Electric Field

**Figure 4:**
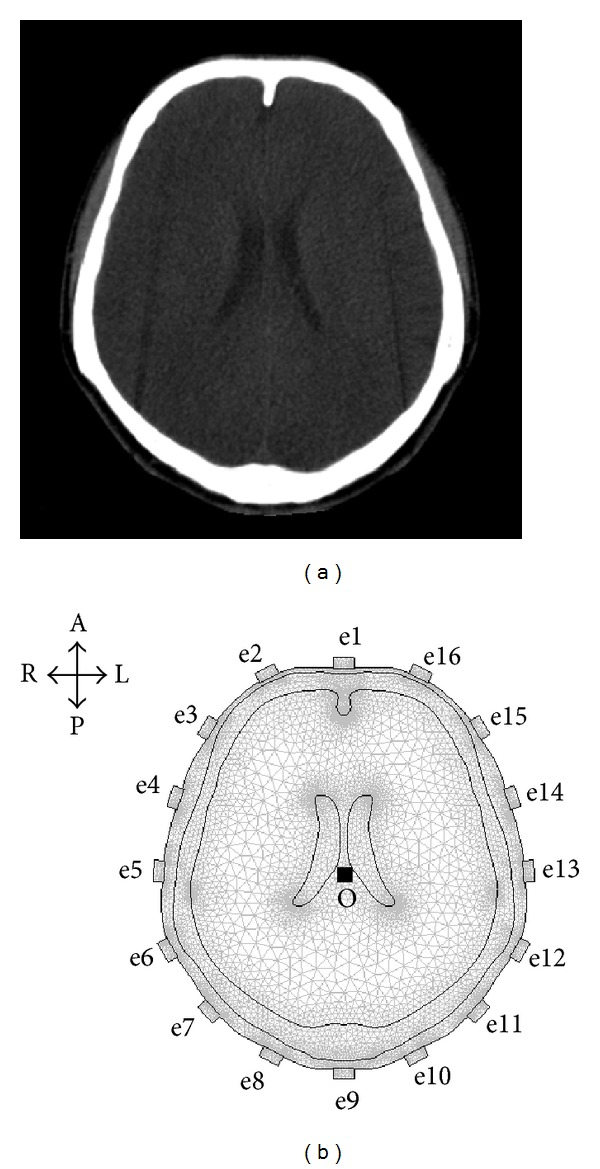
CT image and 2D finite element model of human head used for simulation experiments. (a) A head CT image of a healthy volunteer was used to construct a finite element model. (b) A finite element model (FEM) with an ideally symmetrical structure was constructed according to the right boundary of each layer of head tissues in the head CT image. The 2D head model consisted of 17659 triangular elements, 9200 nodes, and 16 electrodes (A: anterior; P: posterior; L: left; and R: right).

The 2D EIT electrodes are lying on an axial plane approximately 3 cm above inion of the human head; therefore, a head CT image (Figure 4(a)) of a healthy volunteer was utilized to construct a 2D head model. We duplicated the right boundary of each layer of head tissues in the head CT image and mirrored it to the left side to construct a finite element model for the purposes of studying the feasibility of reconstructing the stroke lesion by SEIT. According to finite element modeling [32], a 2D human head finite element model (FEM) with ideally bilateral symmetry was established with COMSOL Multiphysics 3.5a (COMSOL, Inc., Stockholm, Sweden) (Figure 4(b)). The model contained the layers of scalp, skull, brain tissue, and cerebrospinal fluid from the outside to the inside. The conductivity of the layers was set as follows: 0.4400 S/m for the scalp [33], 0.0126 S/m for the skull (the conductivity of standard trilayer skull in the literature [34]), 0.2500 S/m for brain tissues [35], and 1.2500 S/m for cerebrospinal fluid (CSF) [36]. Sixteen rectangles with a width of 1 cm and height of 0.5 cm were set on the outermost layer of the scalp. The material property was set as brass, representing 16 EIT electrodes (Electrodes 1 to 16). The centers of these electrodes were set as e1 to e16. Electrodes 1 and 9 were at frontal and occipital poles of the head, respectively. The midpoint of Lines e1–e9 (Point O) was considered the center of the model. Electrodes 5 and 13 were on the horizontal line through Point O. Then, the distance between electrodes 1 and 5 on the scalp was measured, and the connective line between the two electrodes was equally divided into four parts to determine the locations of electrodes 2, 3, and 4. Similarly, the locations of the remaining electrodes were determined.

**Figure 5:**
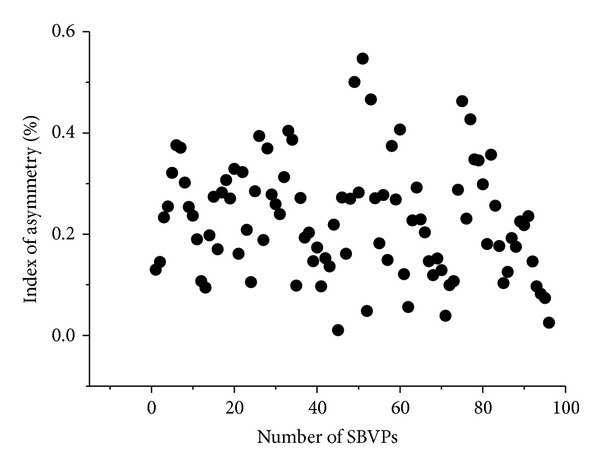
Index of asymmetry (IA) of the 2D FEM of human head.

In one polar drive, the used boundary conditions were as follows: (1) inward current flow (current density = 15.92 A/m^2^) was applied to the outer boundary of electrode 1 to simulate the EIT driving current of 1250 *μ*A, (2) ground was applied to the outer boundary of electrode 9, and (3) all other external boundaries were treated as insulated. In this way, one polar drive simulation was completed. Thus, the electric field formed by the current applied to the head by EIT was simulated. The potential on each electrode was then calculated by solving the Laplace equation, ∇·(*σ*∇*V*) = 0 (*σ*, conductivity; *V*, potential), with the stationary solver of a direct solving method, UMFPACK, in the COMSOL AC/DC Module. The absolute potential differences of the adjacent electrodes were EIT boundary voltages. The remaining 15 polar drives were then simulated according to the EIT driving order. A frame of simulated EIT raw data was obtained after 16 drives. A frame of simulated EIT data was calculated when there was no object in the model, which was utilized to calculate the index of asymmetry (IA) to evaluate the electrical impedance asymmetry of the 2D FEM of human head (Figure 5). The maximum value of IA (IA_max⁡_) was 0.55%.

#### 2.3.2. The Method of SEIT Imaging Based on Simulation Data

**Figure 6:**
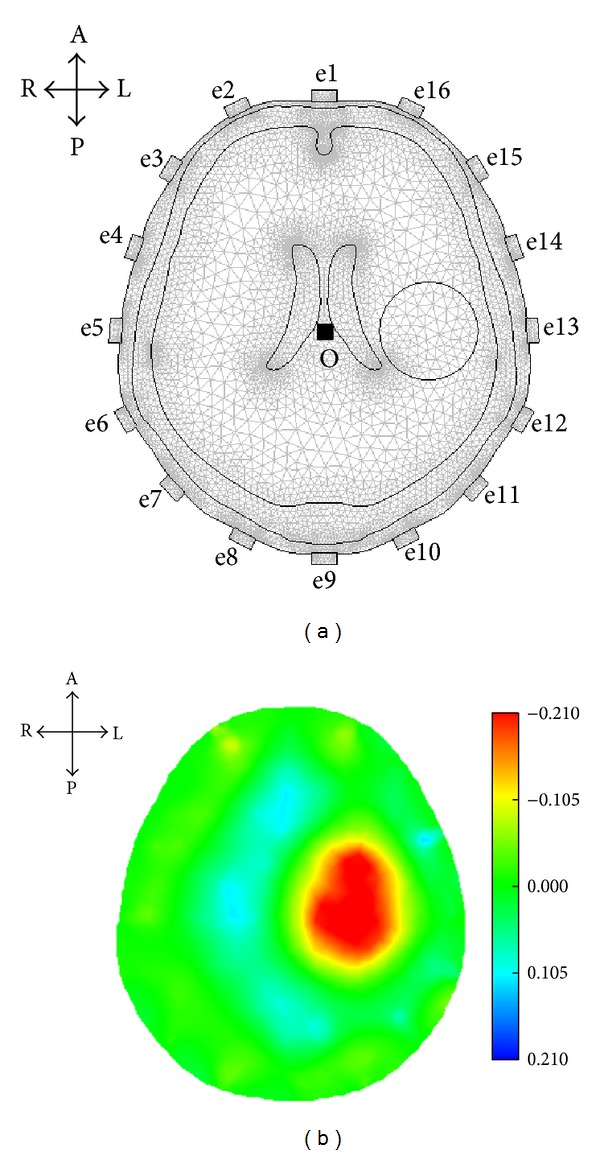
SEIT imaging based on simulated EIT data. (a) A simulated stroke lesion was set on the left side of the model. (b) The reconstructed SEIT image reflected the simulated lesion (A: anterior; P: posterior; L: left; and R: right).

A simulated stroke lesion was set in the model. For example, a circular region was set at the midpoint of line O-e13 of the model, with a conductivity of 0.65 S/m [36] and a radius of 2 cm, to simulate a hemorrhagic stroke lesion (Figure 6(a)); one frame of EIT raw data was then calculated. With half of the frame of EIT raw data measured from the undamaged CCH (the right CCH), one frame of SEIT reference data was constructed. Finally, difference imaging was conducted with EIT raw data and SEIT reference data.

Given the simulated lesion, the maximum absolute value of Δ*ρ* in the image was about 0.210. Thus, Δ*ρ*_max⁡_ in (4) was set as 0.210 to obtain an SEIT image as shown in Figure 6(b). Abnormal resistivity distribution was reconstructed in the SEIT image.

Simulated stroke lesions of different sizes were firstly set in the middle of one hemisphere of the FEM model and the minimum area of the stroke lesion detectable by SEIT was identified. We then verified whether SEIT could reconstruct the stroke lesion in the different lobes of the head model with the identified minimum area. The conductivity of the circular region was set as 0.65 S/m in hemorrhagic stroke lesion modeling; the conductivity of ischemic stroke lesion was set as 0.13 S/m [37] in ischemic stroke lesion modeling.

#### 2.3.3. Simulation-Based SEIT Imaging of Stroke Lesions of Different Sizes

**Figure 7:**
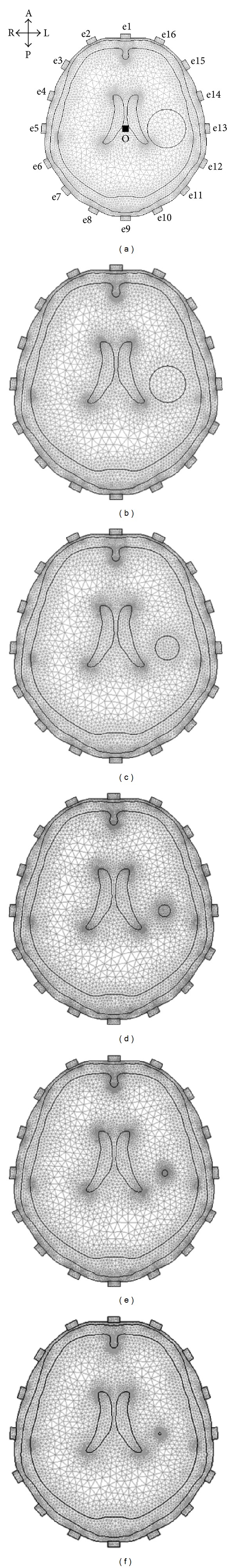
Simulated stroke lesions of different sizes.The radius of the object was set to 2.00, 1.50, 1.00, 0.50, 0.25, and 0.10 cm (from (a) to (f)) (A: anterior; P: posterior; L: left; and R: right). The conductivity of the object was set to 0.65 S/m in the case of simulated hemorrhagic stroke and 0.13 S/m in the case of simulated ischemic stroke.

We successively set a circular region at the midpoint of line O-e13 on the left of FEM, with the radii of 2.00, 1.50, 1.00, 0.50, 0.25, and 0.10 cm, to simulate a hemorrhagic or ischemic stroke lesion of different sizes. The locations and sizes of the simulated stroke lesions are shown in Figure 7. One frame of EIT raw data was obtained in every lesion setting. The corresponding IA_max⁡_ of each frame of EIT data was calculated, and SEIT imaging was conducted. The degree of abnormal resistivity distribution resulted from the object was quantitatively evaluated by calculating MARV of ROI in SEIT images.

#### 2.3.4. Simulation-Based SEIT Imaging of the Stroke Lesion at Different Locations

**Figure 8:**
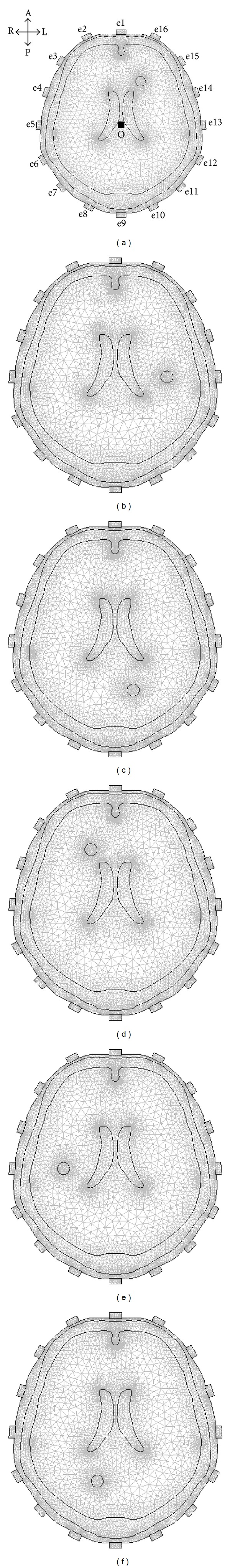
Different locations of simulated stroke lesion set in the model. The radius of the simulated lesion was 0.50 cm. The conductivity of the object was set to 0.65 S/m in the case of simulated hemorrhagic stroke and 0.13 S/m in the case of simulated ischemic stroke (A: anterior; P: posterior; L: left; and R: right).

After the smallest area of stroke lesions detectable by SEIT was identified, we successively set a circular region with the minimal area at the midpoint of lines O-e16, O-e13, and O-e10 on the left of FEM to simulate a hemorrhagic or ischemic stroke lesion in the left frontal lobe, left temporal lobe, and left occipital lobe (Figures 8(a)–8(c)). We also set the same-sized circular region at the midpoint of lines O-e2, O-e5, and O-e8 on the right of FEM to simulate a hemorrhagic or ischemic stroke lesion in the right frontal lobe, right temporal lobe, and right occipital lobe (Figures 8(d)–8(f)). A frame of EIT raw data was obtained after each lesion was set. We also calculated the corresponding IA_max⁡_ of each frame of EIT data and conducted SEIT imaging.

### 2.4. Experiments on SEIT Imaging of the Physical Phantom

#### 2.4.1. Experiment Setups

**Figure 9:**
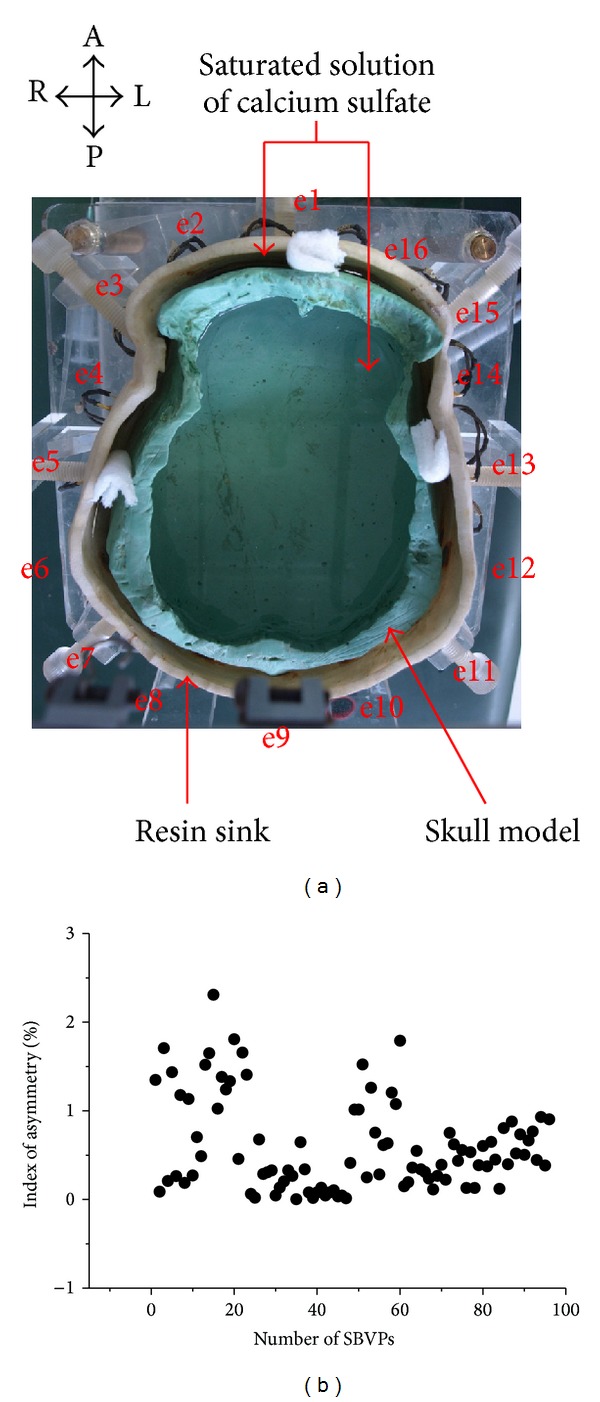
Physical phantom experiments: (a) physical phantom of realistic human head (A: anterior; P: posterior; L: left; and R: right); (b) index of asymmetry (IA) of the physical phantom.

An EIT system named FMEIT-5 [38] was used to measure EIT data. The working frequency of this system is from 1 kHz to 190 kHz with measurement accuracy of ±0.01% and common mode rejection ratio over 80 dB. The system is serial in data acquisition and there are driven screens for the leads connecting the electrodes. The drive current was 1250 *μ*A at 50 kHz. The experiments were conducted with a physical phantom of realistic human head developed by our research group [39], as shown in Figure 9(a). There were three parts, which were a white resin sink, a skull model made of plaster with conductivity of 0.013 S/m, and saturated solution of calcium sulfate with conductivity of 0.2 S/m. The sink and the skull model were made in strict accordance with 3D reconstruction of human head CT, which should guarantee the maximum conformity to real human head in terms of structure and resistivity distribution. Before the experiments, the skull was put on the base of the sink and saturated solution of calcium sulfate was injected into the sink as well as the skull model to simulate scalp and brain tissues. 16 Ag-AgCl electrodes (Electrode 1 to 16) with diameter of 1 cm on the inner wall of the sink were used to collect EIT data. The centers of these electrodes were set as e1 to e16. The locations of the electrodes (Figure 9(a)) are similar to those in the 2D head model (Figure 4(b)). The surface of the solution must be higher than the top of the measurement electrodes by 1 cm. Agar cylinders of 6 cm in height were considered to simulate stroke lesions, of which the radii were 2, 1.5, 1, 0.5, and 0.25 cm, respectively. The conductivity of the agar was 0.65 S/m in the case of simulated hemorrhagic stroke and 0.13 S/m in the case of simulated ischemic stroke. The immersion depth of the agar cylinders into the solution was 5 cm. During the experiments, the room temperature was controlled at 20 ± 1°C.

We first collected data on the physical phantom without agar using EIT for 1 h. After that one frame of EIT data was utilized to calculate the index of asymmetry (IA) to evaluate the electrical impedance asymmetry of the physical phantom (Figure 9(b)). The maximum value of IA (IA_max⁡_) was 2.31%.

**Figure 10:**
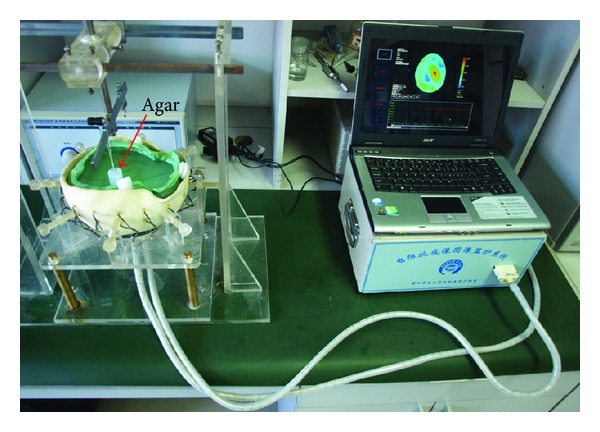
EIT data collection on the physical phantom with an agar cylinder.

Next we put agar cylinders into the physical phantom (the location and size of each agar are shown in the next section) and measured EIT data, as shown in Figure 10. Then SEIT reference data was constructed for each frame of measured EIT data by using the EIT data measured from the hemisphere of the phantom without the agar. SEIT images were acquired by difference imaging method using the measured EIT data and their reference data. The degree of abnormal resistivity distribution in each SEIT image was quantitatively evaluated by calculating MARV of ROI in SEIT images when simulated stroke lesions of different sizes were placed consequently in the phantom.

#### 2.4.2. Placement of Agars in the Physical Phantom

**Figure 11:**
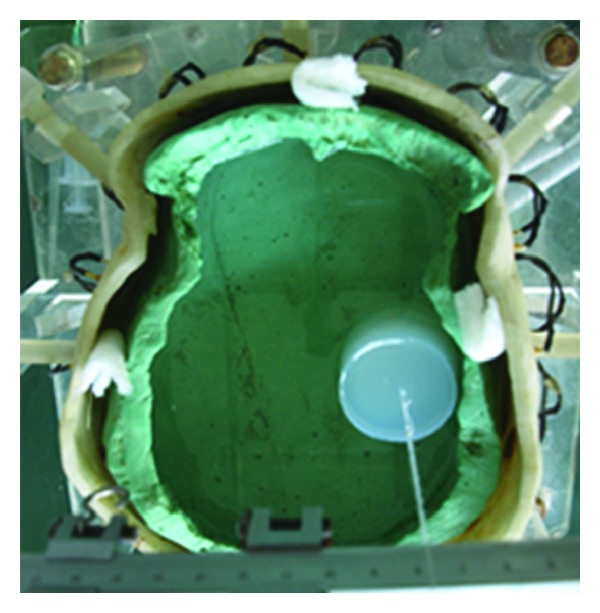
Location of the agar cylinders in the phantom: The initial radius of those cylinders was 2 cm.

*(1) Simulated Stroke Lesions of Different Sizes in the Phantom. *The crossing point of line e1–e9 and line e5–e13 (Point O) was considered the center of the model. Agar cylinders with radii of 2.00, 1.50, 1.00 cm, 0.50 cm, and 0.25 cm were placed at the midpoint of line O-e13 to simulate stroke lesions of different sizes (Figure 11). The conductivity of the agar was 0.65 S/m in the case of simulated hemorrhagic stroke and 0.13 S/m in the case of simulated ischemic stroke. Then the minimum size of simulated stroke lesions detectable by SEIT was determined.

**Figure 12:**
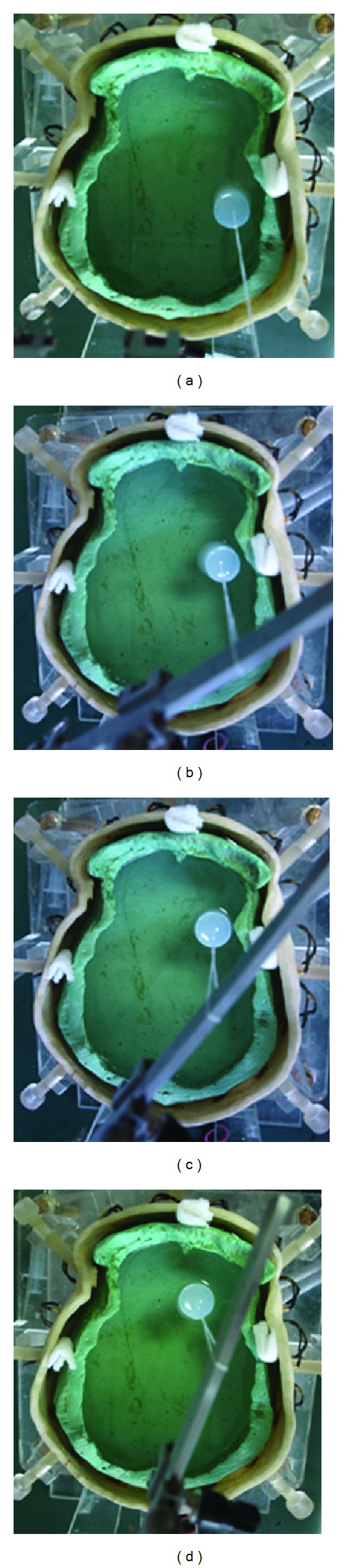
Four locations of the agar cylinder at the anterior and left part of the phantom.

*(2) Simulated Stroke Lesions at Different Locations of the Phantom.* Figure 9(b) indicated that the electrical impedance asymmetry of the anterior part of the phantom is higher than that of the posterior part; therefore, the anterior part of the phantom was used to place agar cylinders to test whether a simulated stroke lesion could be detectable when the head was not well symmetrical. An agar cylinder with a conductivity of 0.65 S/m and a radius of 1 cm (the detected minimum size) was placed at midpoints of the Lines O-e13, O-e14, O-e15, and O-e16 consequently to simulate a hematoma at different locations (Figures 12(a)–12(d)). Then the agar cylinder was placed at symmetrical locations of those four locations (i.e., at midpoints of the lines O-e5, O-e4, O-e3, and O-e2 consequently). Similarly, an agar cylinder with a conductivity of 0.13 S/m was placed at those eight locations to simulate an ischemic lesion at different locations.

## 3. Results and Discussion

### 3.1. Results of Simulation

#### 3.1.1. SEIT Imaging of Stroke Lesions of Different Sizes

**Figure 13:**
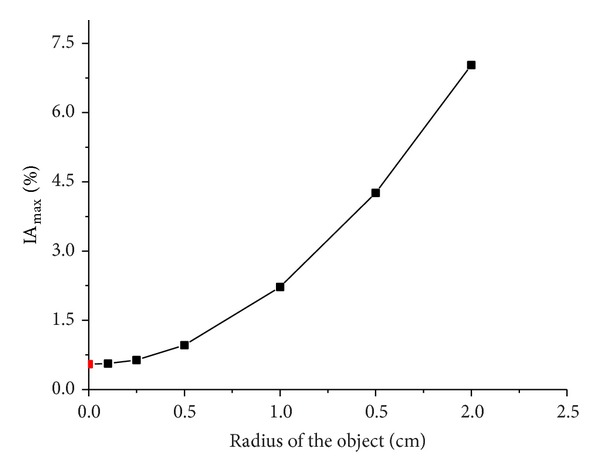
IA_max⁡_ of EIT data from the two hemispheres of the model with and without a hemorrhagic lesion. Black points represent IA_max⁡_ with a simulated hemorrhagic stroke lesion, while the red point represents IA_max⁡_ without a simulated lesion.

**Figure 14:**
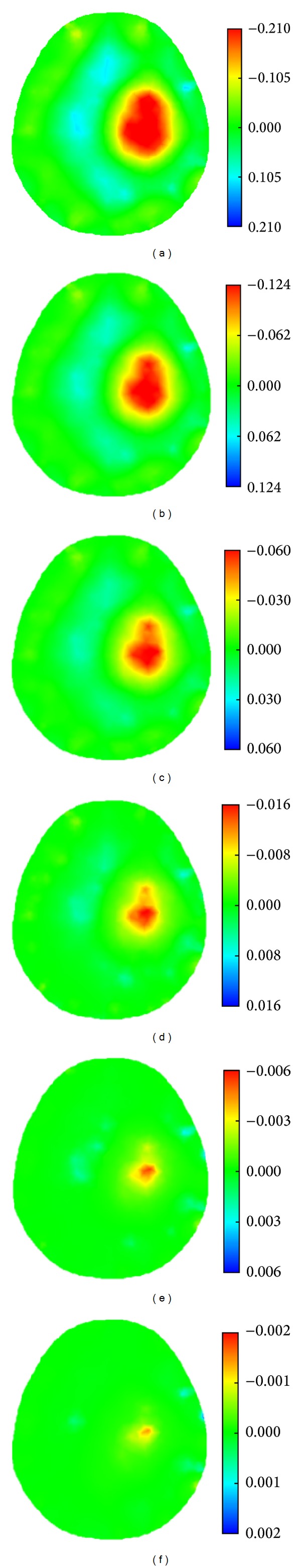
SEIT reconstructions of the simulated hemorrhagic stroke lesions of different sizes. Images (a) to (f) are SEIT reconstructions corresponding to the simulated hemorrhagic stroke lesion with a radius of 2.00, 1.50, 1.00, 0.50, 0.25, and 0.10 cm, respectively.

**Figure 15:**
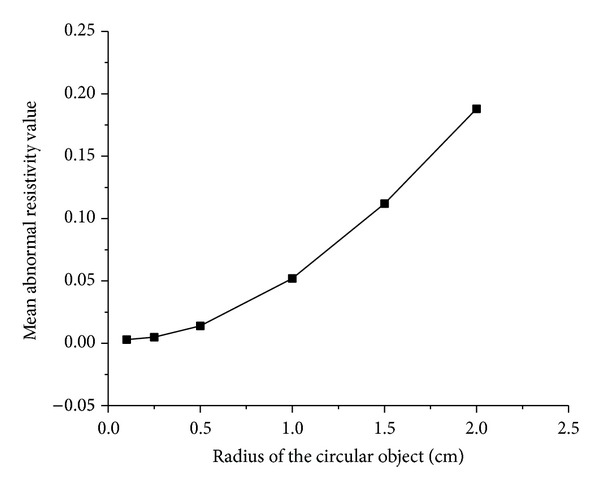
MARV of ROI in the SEIT reconstructions of the simulated hemorrhagic stroke lesions of different sizes: 2.00, 1.50, 1.00, 0.50, 0.25, and 0.10 cm.

When the simulated cerebral hemorrhage lesion gradually decreased (Figures 7(a)–7(f)), the IA_max⁡_ of EIT data from the two hemispheres of the model was gradually reduced (Figure 13); the area of reconstructed object in the SEIT image was also gradually reduced (Figures 14(a)–14(f)). The simulated cerebral hemorrhage lesion on the left of the model exhibited a decrease in resistivity distribution (as the red region) on the left side of the SEIT image. The mean abnormal resistivity value (MARV) also decreased with the decrease in the radius of the simulated hematoma (Figure 15). When the radius of the hemorrhagic lesion was smaller than 0.50 cm, IA_max⁡_ was close to the value with the lesion not set. In these cases, although the SEIT image presented abnormal resistivity distribution, resistivity changed only slightly (<0.006, which can be considered normal).

**Figure 16:**
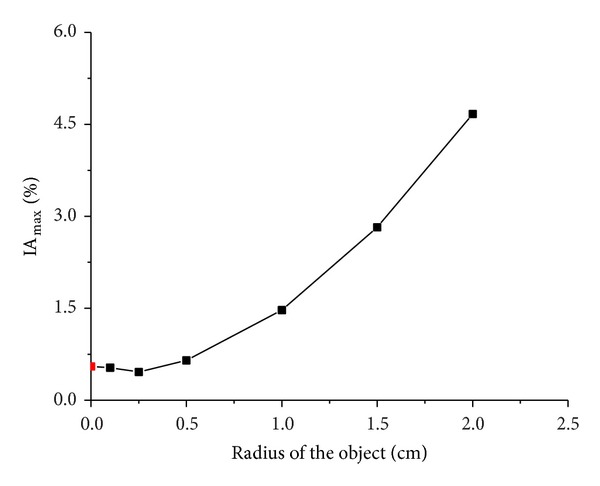
IA_max⁡_ of EIT data from the two hemispheres of the model with and without an ischemic lesion. Black points represent IA_max⁡_ with a simulated ischemic stroke lesion, while the red point represents IA_max⁡_ without a simulated lesion.

**Figure 17:**
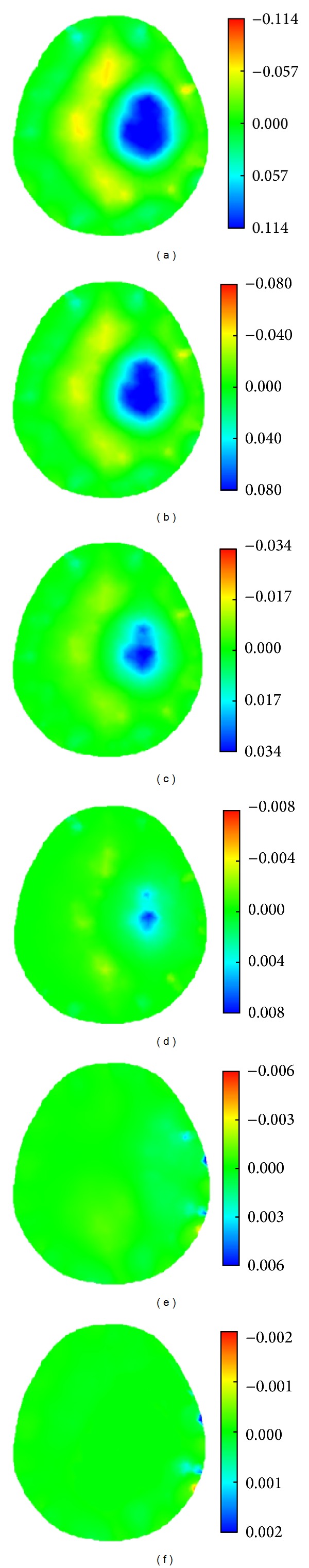
SEIT reconstructions of the simulated ischemic stroke lesions of different sizes. Images (a) to (f) are SEIT reconstructions corresponding to the simulated ischemic stroke lesion with a radius of 2.00, 1.50, 1.00, 0.50, 0.25, and 0.10 cm, respectively.

**Figure 18:**
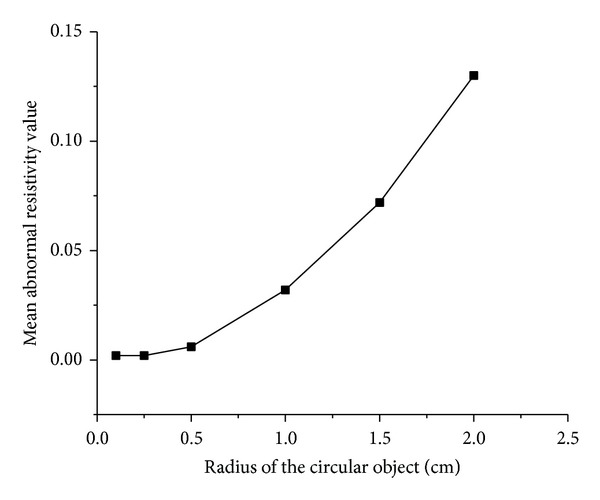
MARV of ROI in the SEIT reconstructions of the simulated ischemic stroke lesions of different sizes: 2.00, 1.50, 1.00, 0.50, 0.25, and 0.10 cm.

When the simulated cerebral ischemia lesion gradually decreased (Figures 7(a)–7(f)), the IA_max⁡_ of EIT data from the two hemispheres of the model gradually reduced (Figure 16); the area of the reconstructed object in the SEIT image also gradually reduced (Figures 17(a)–17(f)). The simulated cerebral ischemic lesion on the left of the model exhibited an increase in resistivity distribution (as the blue region) on the left side of the SEIT image. The MARV also decreased with the decrease in the radius of the simulated hematoma (Figure 18). When the radius of the ischemic lesion was smaller than 0.50 cm, the IA_max⁡_ was close to the value with no lesion set, and the SEIT image did not present an abnormal resistivity distribution.

Hence, according to the simulation imaging of stroke lesions with different sizes, SEIT clearly demonstrated that the minimal radius of the circular simulated stroke lesion was about 0.5 cm. The corresponding SEIT images are as shown in Figures 14(d) and 17(d).

#### 3.1.2. SEIT Imaging of the Stroke Lesion at Different Locations

**Figure 19:**
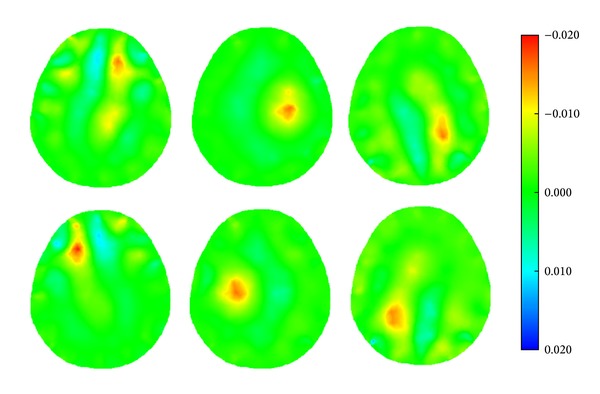
SEIT reconstructions of the simulated hemorrhagic stroke lesion at different locations of the 2D head model.

**Figure 20:**
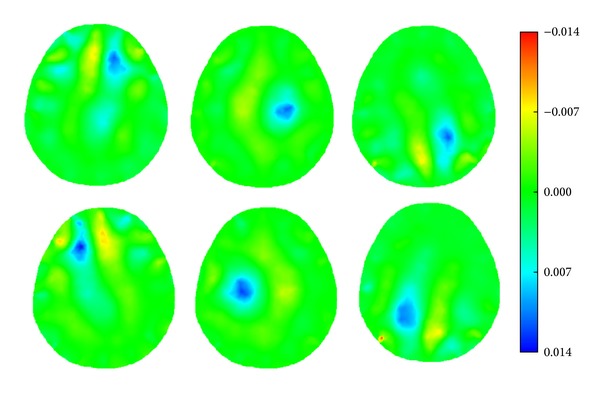
SEIT reconstructions of the simulated ischemic stroke lesion at different locations of the 2D head model.

When a simulated hemorrhagic or ischemic stroke lesion with a radius of 0.50 cm was set at six different locations of the model (Figures 8(a)–8(f)), the image of the hemorrhagic or ischemic stroke lesion was reconstructed by SEIT, as shown in Figures 19 and 20, respectively.

### 3.2. Results of Experiments on the Physical Phantom

#### 3.2.1. Imaging Agar of Different Sizes in the Physical Phantom

**Figure 21:**
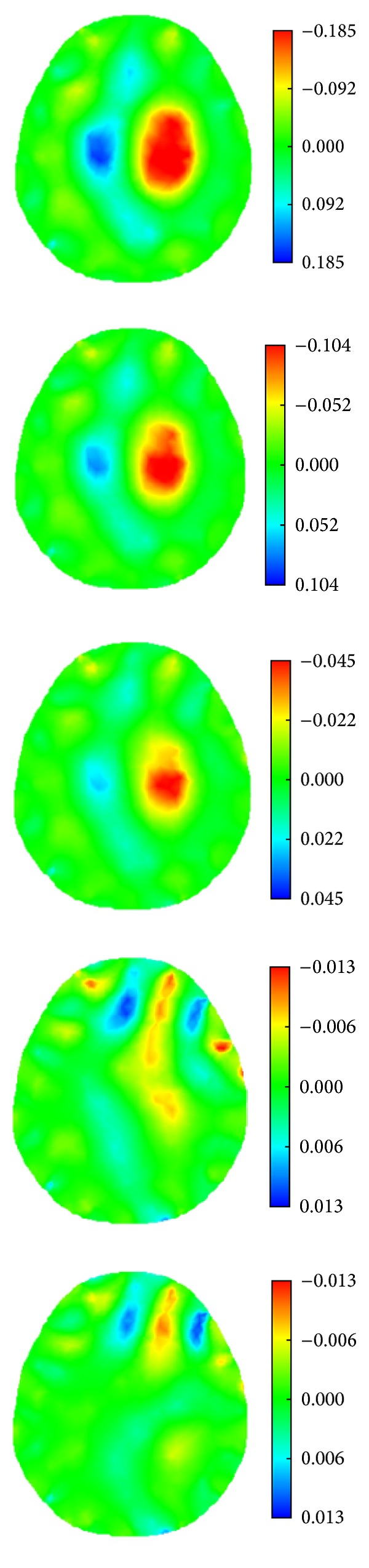
SEIT reconstructions of agar cylinders with radii of 2, 1.5, 1, 0.5, and 0.25 cm to simulate hemorrhagic stroke lesions of different sizes.

**Figure 22:**
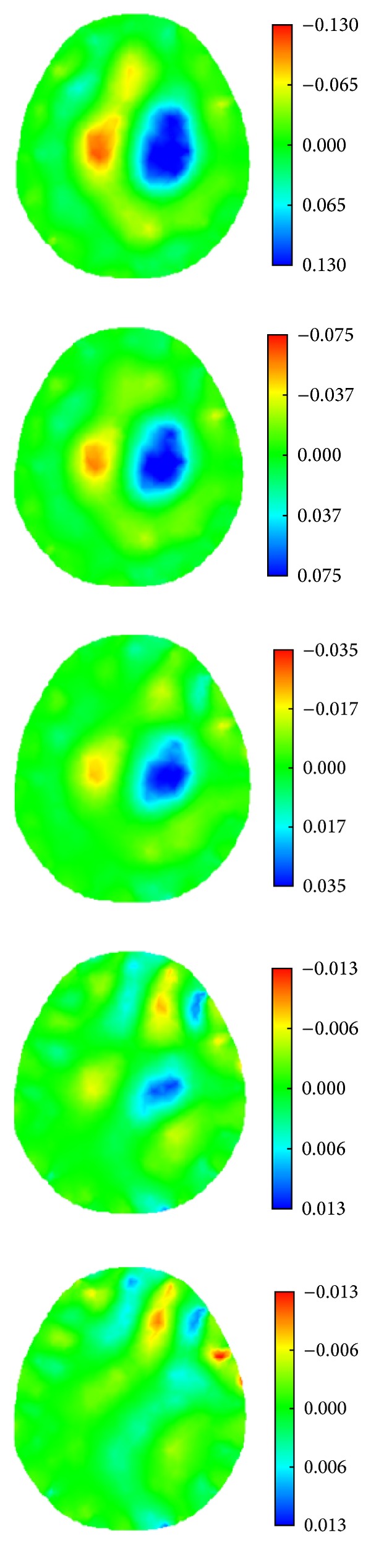
SEIT reconstructions of agar cylinders with radii of 2, 1.5, 1, 0.5, and 0.25 cm to simulate ischemic stroke lesions of different sizes.

**Figure 23:**
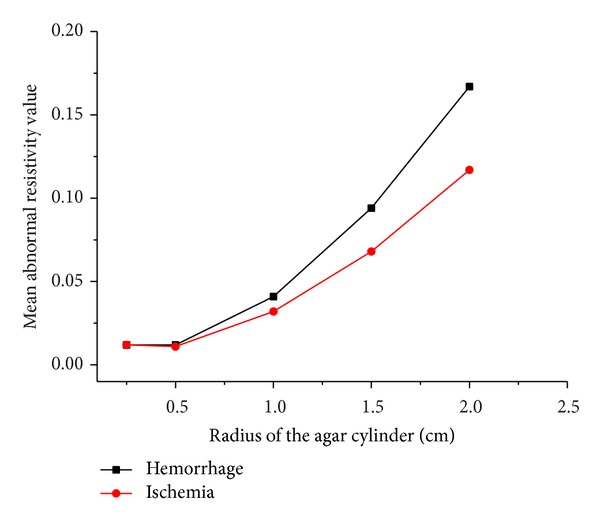
Mean abnormal resistivity value of ROI in each SEIT image of Figures 21 and 22 (hemorrhage for Figure 21 and ischemia for Figure 22).

**Figure 24:**
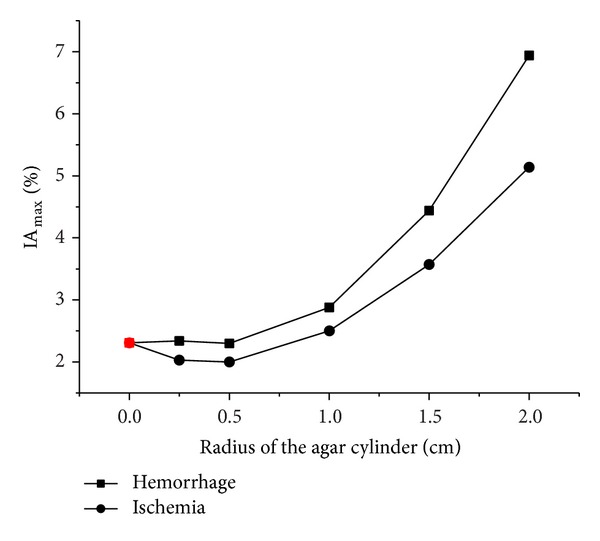
IA_max⁡_ of EIT data from the two hemispheres of the phantom with and without a simulated hemorrhagic or ischemic stroke lesion. Black points represent IA_max⁡_ with a simulated stroke lesion, while the red point represents IA_max⁡_ without a simulated lesion.

When the radius of the agar cylinder for simulating a hemorrhagic or ischemic stroke lesion decreased from 2 cm to 0.25 cm (Figure 11), the area of the reconstructed object also reduced (Figures 21 and 22). The mean abnormal resistivity value (MARV) also decreased with the decrease of the size of agar cylinders (Figure 23). The minimum radius of the agar cylinders detected by SEIT was 1 cm. Those agar cylinders smaller than the minimum size were not reconstructed by SEIT (as shown in the last two images of Figures 21 and 22) and the corresponding maximum values of index of asymmetry (IA_max⁡_) were close to the value with the simulated lesion not set in the phantom (red point in Figure 24).

#### 3.2.2. Imaging Agar at Different Locations of the Phantom

**Figure 25:**
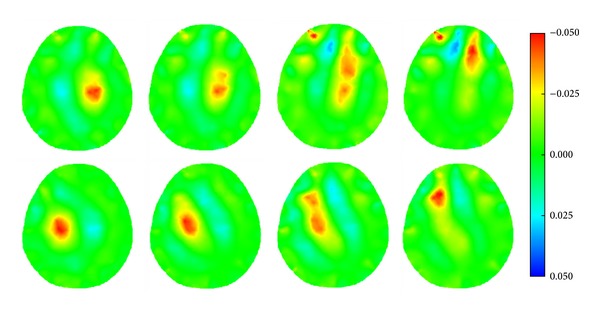
SEIT reconstructions of a simulated hematoma at different locations of the phantom.

**Figure 26:**
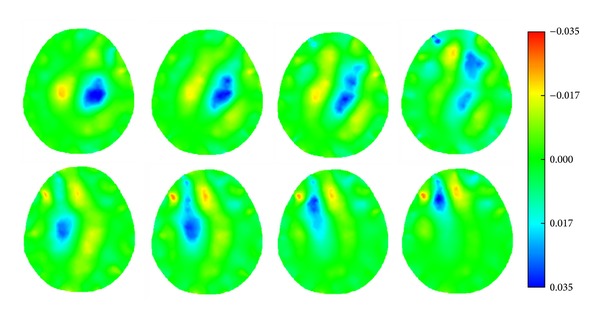
SEIT reconstructions of a simulated ischemic lesion at different locations of the phantom.

When an agar simulating a hematoma or an ischemic lesion (radius: 1 cm) at four different locations of the phantom (Figure 12), the reconstructed SEIT images are in row 1 of Figures 25 and 26, respectively; while the agar cylinder was placed at the symmetrical locations of those in Figure 12, the reconstructed SEIT images are in row 2 of Figures 25 and 26, respectively.

### 3.3. Discussion

#### 3.3.1. Summary of Results

In this work, an SEIT method was proposed to image the difference in resistivity distribution between the two craniocerebral hemispheres (CCHs) of the human head. The significance of this study is that it proposes a novel EIT approach that reconstructs the image of a unilateral cerebral lesion based on one frame of EIT data to provide information on the lesion. Therefore, long-time impedance monitoring adopted by dynamic EIT becomes unnecessary when the method of SEIT is utilized to detection of stroke. Patients with large stroke lesions face high risk of mortality [40] and lesion-led injuries vary with the anatomical location of the lesion [41]. Thus, SEIT should evaluate lesion information on both aspects. The imaging experiments on the 2D head model and those on the physical phantom confirmed that SEIT can use abnormal resistivity distribution to reflect stroke lesions at different locations and with different sizes when the lesion can significantly alter the electrical impedance asymmetry of the head. The electrical impedance asymmetry of the physical phantom was higher than that of the 2D head model; therefore, the detected minimum radius of the objects in the 2D head model was 0.5 cm, which was 1 cm in the physical model. Moreover, the image quality of the physical phantom experiments was lower than that of simulation experiments. Improving the quality of SEIT images is an important issue that needs further study when the imaged object is not well symmetrical.

#### 3.3.2. Possible Limitations of Clinical Application of SEIT

The method of symmetrical electrical impedance tomography (SEIT) reconstructs the images of stroke lesions primarily based on the stroke-induced electrical impedance asymmetry in human heads; therefore, the greater the asymmetry is, the easier the lesion could be detected. This is a notable feature of SEIT. The lesion size of lacunar cerebral infarction is usually smaller than 2 cm in diameter, which is usually found in deep brain. Patients with this kind of infarction may have mild or no symptoms [42]. The minimum size of the simulated cerebral infarcts that could be detected by SEIT in the physical phantom was 2 cm in diameter. Due to the small sizes of the lacunar cerebral infarcts and their deep locations in the brain, the electrical impedance asymmetry in human heads induced by the infarction would be insignificant, which may be ignored by SEIT in clinical application. Imaging experiments on stroke patients by SEIT were not conducted in this study; therefore it was not confirmed whether SEIT could detect lacunar cerebral infarcts in clinical practice. Lacunar cerebral infarcts could be diagnosed by brain CT or MRI currently. They may be detected by SEIT in the future by optimizing the performances of the EIT imaging algorithm and the hardware system.

#### 3.3.3. Further Considerations of This Study

This study is a methodology study to validate the method of SEIT in technique; therefore, simulation and physical phantom experiments were conducted. Preliminary imaging experiments on the heads of stroke patients and normal individuals will be conducted in the future studies to test the feasibility of SEIT in detection of stroke in practice and some important factors should be considered.

It is of great necessity to correlate the neuroimaging data obtained by brain CT and/or MRI with those obtained by SEIT in the further imaging experiments on the heads of stroke patients. The correlation analysis should include location and area of the reconstructed objects by the two kinds of image reconstruction means. In location correlation analysis the central coordinates of the two kinds of reconstructed objects are calculated first [43, 44] and then linear regression analysis is conducted. In area correlation analysis the area of the two kinds of the reconstructed objects are calculated [43, 44] followed by the linear regression analysis. The feasibility of clinical application of SEIT in detection of stroke would be verified by the above analyses.

The symmetrical distribution of EIT electrodes at the two sides of the imaged head by SEIT should be ensured. The asymmetrical placement of electrodes may lead to notable artifacts in reconstructed images. This was not a problem in this study, since the electrodes were precisely located in the 2D head model and the physical phantom. To ensure the symmetrical distribution of electrodes on the head in the further experiments, the location of each electrode should be precisely measured on human subjects before the placement of EIT electrodes. The data collection maybe time-consuming. Some researchers employed the Polhemus FASTRAK digitizer to accurately position electrodes [45], and several other researchers proposed a hydrogel elasticated headnet suitable for EIT data collection by comparing several EIT electrode headnets [46]. Therefore, we will develop a headnet suitable for SEIT imaging to quickly and accurately place EIT electrodes on human heads.

#### 3.3.4. Potential in Determination of Stroke Type by SEIT

Since the symptoms of a hemorrhagic stroke and an ischemic stroke are similar, it is necessary to identify different types of stroke. For instance, thrombolytic therapy must not be given to patients with hemorrhagic stroke, as the hemorrhage may extend. Therefore, identification of stroke type is essential to permit appropriate treatment to stroke patients.

The resistivity of a hemorrhagic lesion is lower than that of normal brain tissue in the contralateral hemisphere, whereas the resistivity of the lesion is higher when cerebral infarction occurs. This was demonstrated by the reconstructions of simulated hemorrhagic and ischemic stroke lesions in the current study. The SEIT images of the two types of simulated stroke lesions were clearly different. Thus, SEIT may characterize the two types of stroke with opposite resistivity changes.

## 4. Conclusion

A new EIT method (SEIT) was proposed to reconstruct the impedance image of unilateral stroke lesions. The preliminary imaging results of the 2D head model and the physical phantom verified the method in rapid detection of unilateral stroke lesions. In our future study, we will perfect this method and conduct experiments on suspected stroke patients in community hospitals to validate the feasibility of SEIT in rapid detection of stroke.

## Figures and Tables

**Table 1:** RGB color mapping for the pixel (*x*, *y*) based on the mapping index *g*(*x*, *y*).

*g*(*x*, *y*)	R	G	B
0.75 < *g*(*x*, *y*) < 1	0	1022 − 1020∗*g*(*x*, *y*)	255
0.5 < *g*(*x*, *y*) < 0.75	0	255	1020∗*g*(*x*, *y*) − 510
0.25 < *g*(*x*, *y*) < 0.5	510 − 1020∗*g*(*x*, *y*)	255	0
0 < *g*(*x*, *y*) < 0.25	255	1020∗*g*(*x*, *y*)	0

**Table 2:** SBVPs from electrode pair (1, 2) and electrode pair (1, 16).

U_3,1_	U_4,1_	U_5,1_	U_6,1_	U_7,1_	U_8,1_	U_11,1_	U_12,1_	U_13,1_	U_14,1_	U_15,1_	U_16,1_
U_15,16_	U_14,16_	U_13,16_	U_12,16_	U_11,16_	U_10,16_	U_7,16_	U_6,16_	U_5,16_	U_4,6_	U_3,16_	U_2,16_
1	2	3	4	5	6	7	8	9	10	11	12

**Table 3:** SBVPs from electrode pair (2, 3) and electrode pair (16, 15).

U_1,2_	U_4,2_	U_5,2_	U_6,2_	U_7,2_	U_8,2_	U_9,2_	U_12,2_	U_13,2_	U_14,2_	U_15,2_	U_16,2_
U_1,15_	U_14,15_	U_13,15_	U_12,15_	U_11,15_	U_10,15_	U_9,15_	U_6,15_	U_5,15_	U_4,15_	U_3,15_	U_2,15_
13	14	15	16	17	18	19	20	21	22	23	24

**Table 4:** SBVPs from electrode pair (3, 4) and electrode pair (15, 14).

U_1,3_	U_2,3_	U_5,3_	U_6,3_	U_7,3_	U_8,3_	U_9,3_	U_10,3_	U_13,3_	U_14,3_	U_15,3_	U_16,3_
U_1,14_	U_16,14_	U_13,14_	U_12,14_	U_11,14_	U_10,14_	U_9,14_	U_8,14_	U_5,14_	U_4,14_	U_3,14_	U_2,14_
25	26	27	28	29	30	31	32	33	34	35	36

**Table 5:** SBVPs from electrode pair (4, 5) and electrode pair (14, 13).

U_1,4_	U_2,4_	U_3,4_	U_6,4_	U_7,4_	U_8,4_	U_9,4_	U_10,4_	U_11,4_	U_14,4_	U_15,4_	U_16,4_
U_1,13_	U_16,13_	U_15,13_	U_12,13_	U_11,13_	U_10,13_	U_9,13_	U_8,13_	U_7,13_	U_4,13_	U_3,13_	U_2,13_
37	38	39	40	41	42	43	44	45	46	47	48

**Table 6:** SBVPs from electrode pair (5, 6) and electrode pair (13, 12).

U_1,5_	U_2,5_	U_3,5_	U_4,5_	U_7,5_	U_8,5_	U_9,5_	U_10,5_	U_11,5_	U_12,5_	U_15,5_	U_16,5_
U_1,12_	U_16,12_	U_15,12_	U_14,12_	U_11,12_	U_10,12_	U_9,12_	U_8,12_	U_7,12_	U_6,12_	U_3,12_	U_2,12_
49	50	51	52	53	54	55	56	57	58	59	60

**Table 7:** SBVPs from electrode pair (6, 7) and electrode pair (12, 11).

U_1,6_	U_2,6_	U_3,6_	U_4,6_	U_5,6_	U_8,6_	U_9,6_	U_10,6_	U_11,6_	U_12,6_	U_13,6_	U_16,6_
U_1,11_	U_16,11_	U_15,11_	U_14,11_	U_13,11_	U_10,11_	U_9,11_	U_8,11_	U_7,11_	U_6,11_	U_5,11_	U_2,11_
61	62	63	64	65	66	67	68	69	70	71	72

**Table 8:** SBVPs from electrode pair (7, 8) and electrode pair (11, 10).

U_1,7_	U_2,7_	U_3,7_	U_4,7_	U_5,7_	U_6,7_	U_9,7_	U_10,7_	U_11,7_	U_12,7_	U_13,7_	U_14,7_
U_1,10_	U_16,10_	U_15,10_	U_14,10_	U_13,10_	U_12,10_	U_9,10_	U_8,10_	U_7,10_	U_6,10_	U_5,10_	U_4,10_
73	74	75	76	77	78	79	80	81	82	83	84

**Table 9:** SBVPs from electrode pair (8, 9) and electrode pair (10, 9).

U_2,8_	U_3,8_	U_4,8_	U_5,8_	U_6,8_	U_7,8_	U_10,8_	U_11,8_	U_12,8_	U_13,8_	U_14,8_	U_15,8_
U_16,9_	U_15,9_	U_14,9_	U_13,9_	U_12,9_	U_11,9_	U_8,9_	U_7,9_	U_6,9_	U_5,9_	U_4,9_	U_3,9_
85	86	87	88	89	90	91	92	93	94	95	96
